# A multifunctional enolase mediates cytoadhesion and interaction with host plasminogen and fibronectin in *Mycoplasma hyorhinis*

**DOI:** 10.1186/s13567-022-01041-0

**Published:** 2022-03-25

**Authors:** Jia Wang, Yanfei Yu, Yao Li, Shiyang Li, Li Wang, Yanna Wei, Yuzi Wu, Bala Pillay, Ademola Olufolahan Olaniran, Thamsanqa E. Chiliza, Guoqing Shao, Zhixin Feng, Qiyan Xiong

**Affiliations:** 1 Institute of Veterinary Medicine, Key Laboratory of Veterinary Biological Engineering and Technology, Ministry of Agriculture and Rural Affairs, Jiangsu Academy of Agricultural Sciences, Nanjing, China; 2 College of Agriculture, Engineering & Science, University of KwaZulu-Natal, Durban, South Africa; 3 School of Life Sciences, Jiangsu University, Zhenjiang, China; 4 College of Veterinary Medicine, Hunan Agricultural University, Changsha, China

**Keywords:** *Mycoplasma hyorhinis*, virulence factor, enolase, adhesion, plasminogen, fibronectin, moonlighting protein

## Abstract

*Mycoplasma hyorhinis* may cause systemic inflammation of pigs, typically polyserositis and arthritis, and is also associated with several types of human cancer. However, the pathogenesis of *M. hyorhinis* colonizing and breaching the respiratory barrier to establish systemic infection is poorly understood. Glycolytic enzymes are important moonlighting proteins and virulence-related factors in various bacteria. In this study, we investigated the functions of a glycolytic critical enzyme, enolase in the infection and systemic spread of *M. hyorhinis*. Bacterial surface localization of enolase was confirmed by flow cytometry and colony hybridization assay. Recombinant *M. hyorhinis* enolase (rEno) was found to adhere to pig kidney (PK-15) cells, and anti-rEno serum significantly decreased adherence. The enzyme was also found to bind host plasminogen and fibronectin, and interactions were specific and strong, with dissociation constant (*K*_D_) values of 1.4 nM and 14.3 nM, respectively, from surface plasmon resonance analysis. Activation of rEno-bound plasminogen was confirmed by its ability to hydrolyze plasmin-specific substrates and to degrade a reconstituted extracellular matrix. To explore key sites during these interactions, C-terminal lysine residues of enolase were replaced with leucine, and the resulting single-site and double-site mutants show significantly reduced interaction with plasminogen in far-Western blotting and surface plasmon resonance tests. The binding affinities of all mutants to fibronectin were reduced as well. Collectively, these results imply that enolase moonlights as an important adhesin of *M. hyorhinis*, and interacts with plasminogen and fibronectin. The two lysine residues in the C-terminus are important binding sites for its multiple binding activities.

**Supplementary Information:**

The online version contains supplementary material available at 10.1186/s13567-022-01041-0.

## Introduction

*Mycoplasma* species (class Mollicutes) are distinguished by their small size, minute genomes, and lack of cell wall. They are widespread in various hosts and cause chronic infections in many cases. *Mycoplasma hyorhinis* is one of four important mycoplasma species considered pathogenic to swine [1]. It is prevalent in pig farms worldwide, and it mainly inhabits the mucosa of the upper respiratory tract and tonsils. Although most infected pigs show no obvious clinical signs, in some cases *M. hyorhinis* can induce severe systemic infection leading to pleuritis, peritonitis, pericarditis, arthritis, eustachitis, and other ailments [1–3]. Besides inducing systemic inflammation in pigs, *M. hyorhinis* has also been linked to human cancers such as gastric, lung, and colon cancers [4, 5]. However, whether *M. hyorhinis* can be transmitted from pigs to humans, potential transmission routes, and the infection proportion in the population remain unknown. Due to the high prevalence of *M. hyorhinis* in pig herds, its potential threat to human health cannot be ignored, and relevant epidemiology and pathogenic mechanism research is urgently needed.

Mycoplasmas are membrane-associated pathogens, hence adhesion factors are usually considered to be the most important virulence factors. The variable lipoprotein (Vlp) family, P37, and glyceraldehyde-3-phosphate dehydrogenase (GAPDH) [6–8] are the only adhesion molecules of *M. hyorhinis* identified to date, and many more likely remain to be revealed. Systemic spreading of *M. hyorhinis* from the site of infection to the rest of the body is assumed to be critical for disease progression, but the associated mechanism remains unclear [1].

In our previous work, GAPDH, a key enzyme in the glycolysis pathway, was found to be a plasminogen receptor (PlgR) of *M. hyorhinis* that hijacks plasminogen on the bacterial surface [8]. It probably helps to arm *M. hyorhinis* with a proteolytic activity to degrade tissue barriers such as extracellular matrix (ECM) and fibrin clots. Enolase is another well-known bacterial PlgR. This key glycolytic enzyme catalyzes the conversion of 2-phosphoglycerate to phosphoenolpyruvate. Besides exerting enzyme activity in the cytoplasm, enolase can also be present at the surface of many bacteria to play other roles during infection, as demonstrated for streptococci [9], *Salmonella* Typhimurium [10], *Mycobacterium tuberculosis* [11], *Klebsiella pneumoniae* [12], as well as some eukaryotic parasites such as *Taenia solium* [13] and *Leishmania mexicana* [14].

In the present study, we explored non-canonical functions of enolase in cytoadhesion of *M. hyorhinis*, and investigated interactions with plasminogen and fibronectin host molecules.

## Materials and methods

### Preparation of recombinant enolase protein and its site-directed mutants

The full-length gene encoding *M. hyorhinis* enolase (strain HUB-1, GenBank, CP002170.1, MHR_0469) was codon-optimized and synthesized (GenScript, China). Meanwhile, three different site-directed mutants in which C-terminal lysine residues were replaced with leucine were generated using a Mut Express MultiS Fast Mutagenesis Kit (Vazyme, China). They included two distinct single point mutations (residues 451 or 452) and one double mutation (residues 451 and 452). All open reading frames (ORFs) were cloned into pET-28a ( +) and introduced into *Escherichia coli* strain BL21 (DE3). *E. coli* cells in the early log phase were induced with 1 mM isopropyl-beta-D-thiogalactopyranoside (IPTG) and cultured at 18 °C overnight. Recombinant enolase proteins were purified from ultrasonic bacterial supernatants by nickel affinity chromatography (GenScript), then concentrated and buffer-exchanged by ultrafiltration using a Centricon (Millipore, USA). Recombinant enolase proteins were denoted as rEno wild-type (WT), K451L (single point mutation at residue 451), K452L (single point mutation at residue 452) and K451L-K452L (double mutation of residues 451 and 452).

### Preparation of polyclonal antibody against *M. hyorhinis* enolase

Polyclonal antibody against *M. hyorhinis* enolase was obtained by immunizing 4-week-old New Zealand white rabbits with WT rEno protein. Purified rEno was emulsified with complete (first immunity) and incomplete (second to fourth immunity) Freund adjuvant (1:1, v/v) and subcutaneously immunized four times at 2-week intervals. Antisera were collected 2 weeks after the fourth immunization and titers were determined by enzyme-linked immunosorbent assay (ELISA) [15]. Briefly, 96-well plates were coated with 10 μg/mL WT rEno protein overnight at 4 °C. After blocking with 5% BSA, twofold serially diluted anti-rEno serum and preimmune serum were added to wells and incubated for 30 min at 37 °C, followed by 100 µL goat anti-rabbit IgG-HRP at a dilution of 1:10 000. The optical density (OD) value was measured at 450 nm. The maximum antibody dilution that fulfilled the criteria (OD_positive_/OD_negative_ > 2.1) was considered as the antibody titer. The specificity of the prepared polyclonal antibody was assessed by Western blotting (Additional file 1).

### Detection of surface exposed enolase in *M. hyorhinis*

Flow cytometry was used to detect whether enolase was displayed on the surface of *M. hyorhinis* cells. *M. hyorhinis* strain HUB-1, kindly provided by Prof. Shaobo Xiao from Huazhong Agricultural University, China, was cultured in KM2 medium (a modified Friis medium) containing 20% (v/v) swine serum at 37 °C. *M. hyorhinis* cells (1 × 10^7^ color change unit (CCU) / mL) were washed twice with phosphate-buffered saline (PBS) before incubation with rabbit anti-rEno serum at a 1:100 dilution. The preimmune serum was used as a negative control and PBS served as a blank control. Goat anti-rabbit IgG–FITC antibody (1:500 dilution) was used as a secondary antibody. Mycoplasma cells were detected using a BD Accuri C6 flow cytometer (BD Biosciences, USA), and assays were performed in triplicate.

Additionally, the colony blot technique was used to confirm the presence of enolase at the surface of *M. hyorhinis* colonies. The experiment was conducted under conditions without damaging the *M. hyorhinis* cell membrane. A polyvinylidene fluoride (PVDF) membrane was gently placed on mycoplasma colonies on the surface of agar plates. After 5 min, the PVDF membrane was removed, blocked for 2 h at 37 °C with TBST (20 mM Tris, 140 mM NaCl, 0.1% Tween-20) containing 5% skim milk, and incubated overnight at 4 °C in TBST containing anti-rEno serum (1:1000 dilution). The PVDF membrane was then washed with TBST and treated with horseradish peroxidase (HRP)-conjugated goat anti-rabbit IgG (1:10 000 dilution; Boster, China) for 1 h at 37 °C. Finally, the membrane was developed with ECL substrate (Tanon, China) using a ChemiDoc XRS + system. Preimmune serum was used instead of anti-rEno serum as a negative control.

### Adhesion of rEno to PK-15 cells

The indirect immunofluorescence assay was used to determine whether rEno could adhere to the surface of PK-15 cells, a porcine epithelial cell line derived from a normal pig kidney. PK-15 cells were propagated in a 24-well cell culture dish for 24 h. After three washes with PBS, 100 μg rEno was added and incubated with cells for 1 h at 37 °C. Rabbit anti-rEno serum (1:500 dilution) was used as primary antibody and goat anti-rabbit FITC-IgG (1:500 dilution) was used as secondary antibody. Finally, cell nuclei were stained with 4',6-diamidino-2-phenylindole dihydrochloride (DAPI, Beyotime Biotechnology, China) and immunofluorescence was detected using a Zeiss Axiovert fluorescence microscope (Zeiss, Germany). Bovine serum albumin (BSA) replaced rEno as a negative control.

### Inhibition of *M. hyorhinis* cytoadhesion by anti-Eno antibodies

An adhesion inhibition assay was used to study the role of enolase in *M. hyorhinis* adhesion to PK-15 cells. Cells were seeded in 24-well plates and cultured to confluence. Before the adhesion assay, freshly cultured *M. hyorhinis* cells were harvested and incubated with rabbit anti-rEno serum or preimmune serum (1:20 dilution) for 30 min at 37 °C before interacting with cells. After washing three times, *M. hyorhinis* cells (1 × 10^7^ CCU, MOI = 20) suspended in RPMI-1640 medium were added to PK-15 cell-containing wells and incubated at 37 °C for 2 h. The dissociated *M. hyorhinis* cells were removed by washing three times with PBS, and cells in wells were digested with 0.25% trypsin and collected. The mixture was subjected to bacterial genome extraction and real-time PCR [16] for bacterial counting. Assays were performed in triplicate.

### Binding activities of recombinant enolase protein to host plasminogen and fibronectin

The microtiter plate adhesion assay (MPAA) was used to detect binding between rEno and plasminogen (Sigma-Aldrich, USA) or fibronectin (Sigma-Aldrich). For this, 96-well microtiter plates were coated with 100 μL plasminogen or fibronectin (10 μg/mL) overnight at 4 °C. After blocking with 5% BSA, 100 μL of various concentrations of rEno or PBS were added to each well and incubated at 37 °C for 2 h. After washing, binding was evaluated by adding 100 μL of rabbit anti-rEno serum (1:1000 dilution). Next, 100 μL of HRP-conjugated goat anti-rabbit IgG (1:10 000 dilution) was added and incubated at 37 °C for 1 h. After washing, 100 μL of substrate containing 3,3',5,5'-tetramethylbenzidine (TMB) and H_2_O_2_-urea was added and the plate was incubated at room temperature for 10 min. The reaction was stopped by H_2_SO_4_, the absorbance was measured at 450 nm, and all experiments were performed in triplicate.

### Comparing the WT and mutant enolase molecules in their abilities to bind plasminogen and fibronectin

The abilities of WT and mutant enolase enzymes to bind plasminogen and fibronectin were first compared by far-Western blotting. Recombinant proteins (20 μg) were respectively separated by 12% SDS-PAGE and transferred to a PVDF membrane. BSA was used as a negative control. After blocking with 5% skim milk overnight at 4 °C, the membrane was incubated for 1 h at 37 °C with 10 μg/mL plasminogen or fibronectin. Anti-plasminogen (1:1000 dilution, Boster, China) or anti-fibronectin (1:1000 dilution, Beyotime Biotechnology, China) was then incubated with the membrane for 1 h at 37 °C, followed by incubation with HRP-conjugated secondary antibody (1:10 000 dilution), and the membrane was washed with TBST and developed with ECL substrate.

To further characterize the interactions of recombinant proteins with plasminogen and fibronectin, binding parameters were investigated by surface plasmon resonance (SPR) analysis. Plasminogen and fibronectin were separately diluted to 50 μg/mL and covalently immobilized on CM5 sensor chips via amine coupling (Biacore AB, China). Immobilization of soluble plasminogen and fibronectin generated resonance units (RU) of 600. Recombinant proteins were serially diluted (0–4000 nM) in running buffer comprising 10 mM HEPES, 150 mM NaCl, 3 mM EDTA, and 0.05% (v/v) surfactant P20, and injected at a 30 μL/min flow rate for 180 s at 20 °C. The dissociation phase was monitored for 1000 s by allowing buffer to flow over the chip. Kinetic parameters were calculated using Biacore X100 Control software (General Electric, USA).

### Activation of rEno-bound plasminogen and ECM degradation

Next, 96-well microtiter plates were coated with rEno protein (30 μg/mL, 100 µL/well) overnight at 4 °C. After blocking with 5% BSA, plates were incubated for 3 h at 37 °C with 100 μL of plasminogen (10 μg/mL) in the presence or absence of 200 mM ε-aminocaproic acid (ε-ACA; Sigma-Aldrich), a lysine analogue. After washing, 100 μL of tissue-specific plasminogen activator (tPA; Sigma-Aldrich) was diluted to 500 ng/mL and incubated at 37 °C for 2 h. After washing, the plasmin-specific substrate D-Val-Leu-Lys p-nitroanilide dihydrochloride (Sigma-Aldrich) was added at a final concentration of 0.4 mM and incubated at 37 °C. After 24 h, the activity was estimated from the OD value measured at 405 nm. Wells without adding tPA were set to determine the ability of rEno protein to directly activate plasminogen.

Activation was further determined in the solution state. rEno (20 μg/mL) and plasminogen (20 μg/mL) were mixed and incubated for 1 h at 37 °C, and the mixture was added to a flat-bottomed 96-well plate. Next, 500 ng/mL of tPA was added to wells and incubated for 15 min, and 0.4 mM of substrate was added and the plate was incubated at 37 °C. The OD value was measured at 405 nm every 15 min from 15 min until 120 min. Wells containing only plasminogen with tPA, or rEno with plasminogen, or rEno with tPA served as controls. Experiments were performed in triplicate.

The ability of the resulting rEno-bound plasmin to degrade a commercial ECM (Matrigel, Corning, USA) was assessed by scanning electron microscopy (SEM). Firstly, Matrigel was diluted 1:3 in ice-cold PBS, and this was layered on a 3 μm transparent membrane insert (Corning) and allowed to gel for 30 min at 4 °C, then dried overnight at 37 °C. The gel was rehydrated with 70 μL PBS for 1 h at 37 °C before proceeding with the degradation assay. Polystyrene beads were used to immobilize proteins. The rEno or BSA was passively adsorbed on the particle surface according to the manufacturer’s instructions. Briefly, 1% (v/v) beads were suspended in 1 mL of rEno or BSA solution (1.5 mg/mL) and incubated overnight at 4 °C. After washing, beads were blocked with 5% BSA. Subsequent to PBS washing, beads were incubated with 10 μg/mL of plasminogen for 3 h at 37 °C. Thereafter, beads were washed with sterile PBS to remove unbound plasminogen. After incubation with tPA (500 ng/mL) for an additional 2 h at 37 °C, beads were washed and resuspended in 1 mL PBS. The resuspended beads were added to the upper compartment of the transwell, while the lower compartment contained 700 μL PBS. The culture chambers were incubated at 37 °C for 40 h. The obtained transwell membrane was fixed with 2.5% glutaraldehyde and subjected to SEM using Zeiss EVO-LS10 SEM instrument (Zeiss, Germany).

### Statistical analysis

The data of flow cytometry and *M. hyorhinis* cytoadhesion inhibition were analyzed by Student *t*-tests. The data of the enhancement of rEno on plasminogen activation was analyzed by repeated measures ANOVA. Other data were analyzed by one-way ANOVA. *p*-values < 0.05 were considered statistically significant.

## Results

### Cellular surface localization of *M. hyorhinis* enolase

**Figure 1:**
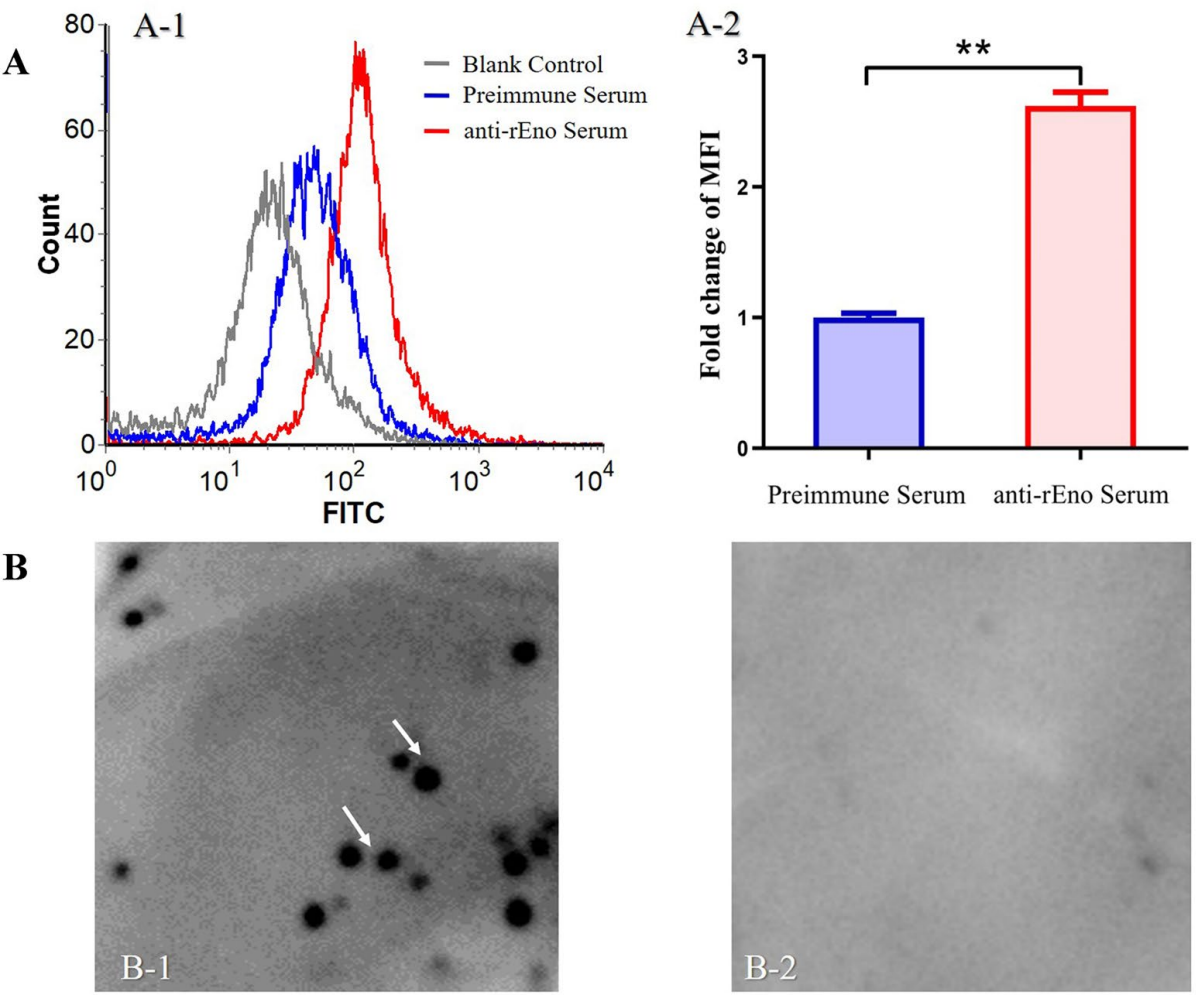
**Detection of enolase on the surface of** ***M. hyorhinis***. **A** Flow cytometry analysis of the surface localization of enolase. Blank control, *M. hyorhinis* cells treated with PBS; Negative control, *M. hyorhinis* cells treated with preimmune serum; anti-rEno, *M. hyorhinis* cells treated with anti-rEno serum (**A-1**). The mean fluorescence intensity (MFI) of *M. hyorhinis* incubated with anti-rEno serum is expressed as the percentage of the corresponding strain incubated with preimmune serum. Results are expressed as mean ± standard deviation (SD) of three experiments with triplicate samples. Asterisks above charts indicate statistically significant differences (**p < 0.01; **A-2**). **B** Colony blot analysis of the surface localization of enolase. Immunostaining with anti-rEno serum (**B-1**) or preimmune serum (**B-2**) was performed after transferring *M. hyorhinis* colonies to a PVDF membrane.

Flow cytometry analysis and colony hybridization assays were conducted to evaluate whether enolase is located on the surface of *M. hyorhinis*. As shown in Figure 1A, significant fluorescence was detected in *M. hyorhinis* cells incubated with rabbit anti-rEno serum. The mean fluorescence intensity (MFI) of the group treated with anti-rEno serum was > 2.5-fold higher than that of the group treated with preimmune serum. This result indicates that enolase was expressed on the surface of *M. hyorhinis* cells.

The surface location was further confirmed by colony immunological hybridization without damaging the cell membrane. Freshly harvested *M. hyorhinis* colonies from the surface of agar plates were transferred to a PVDF membrane. Positive hybridization dots were observed after hybridization with anti-rEno serum, but no reaction was observed after treating with preimmune serum (Figure 1B).

### Enolase contributes to *M. hyorhinis* adhesion to PK-15 cells

**Figure 2:**
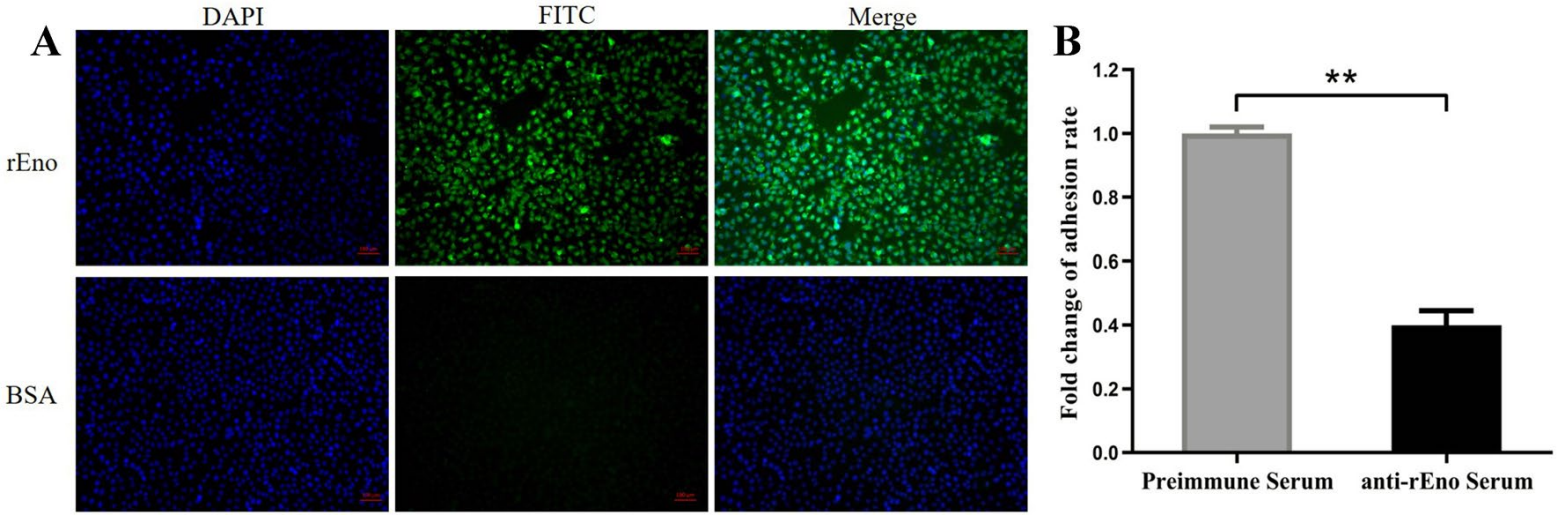
**Cytoadhesive function of *M. hyorhinis*** **enolase.** **A** Cytoadhesion of rEno detected by indirect immunofluorescence assay. rEno or BSA was incubated with PK-15 cells. Bound protein was detected with anti-rEno serum and FITC-conjugated secondary antibody (green). PK-15 cell nuclei were stained with DAPI. **B** Adhesion inhibition assay of anti-rEno antibody. The fold change in adhesion rate is (the number of bacteria adhering to cells incubated with anti-rEno serum / the number of bacteria adhering to cells incubated with preimmune serum) × 100%. Results are expressed as means ± SD of three experiments with triplicate samples (**p < 0.01).

Cytoadhesion is a critical step in mycoplasma infection. The cytoadhesive function of *M. hyorhinis* enolase was investigated by indirect immunofluorescence assay of swine PK-15 cells, and the results were visualized by fluorescence microscopy. As shown in Figure 2A, bright green fluorescence was observed in cells incubated with rEno, whereas no significant fluorescence was observed in control cells incubated with BSA. This result demonstrates that rEno specifically binds to PK-15 cell membranes.

To further evaluate the contribution of enolase to the cytoadhesion of *M. hyorhinis*, adhesion inhibition assays were performed. After blocking with anti-rEno serum, the adherence ability of *M. hyorhinis* to PK-15 cells decreased by 60% compared with the control group treated with preimmune serum (Figure 2B). These results indicate that enolase functions as an important adhesin molecule on the *M. hyorhinis* cell membrane surface.

### Enolase acts as a receptor for *M. hyorhinis* binding to host plasminogen and fibronectin

**Figure 3:**
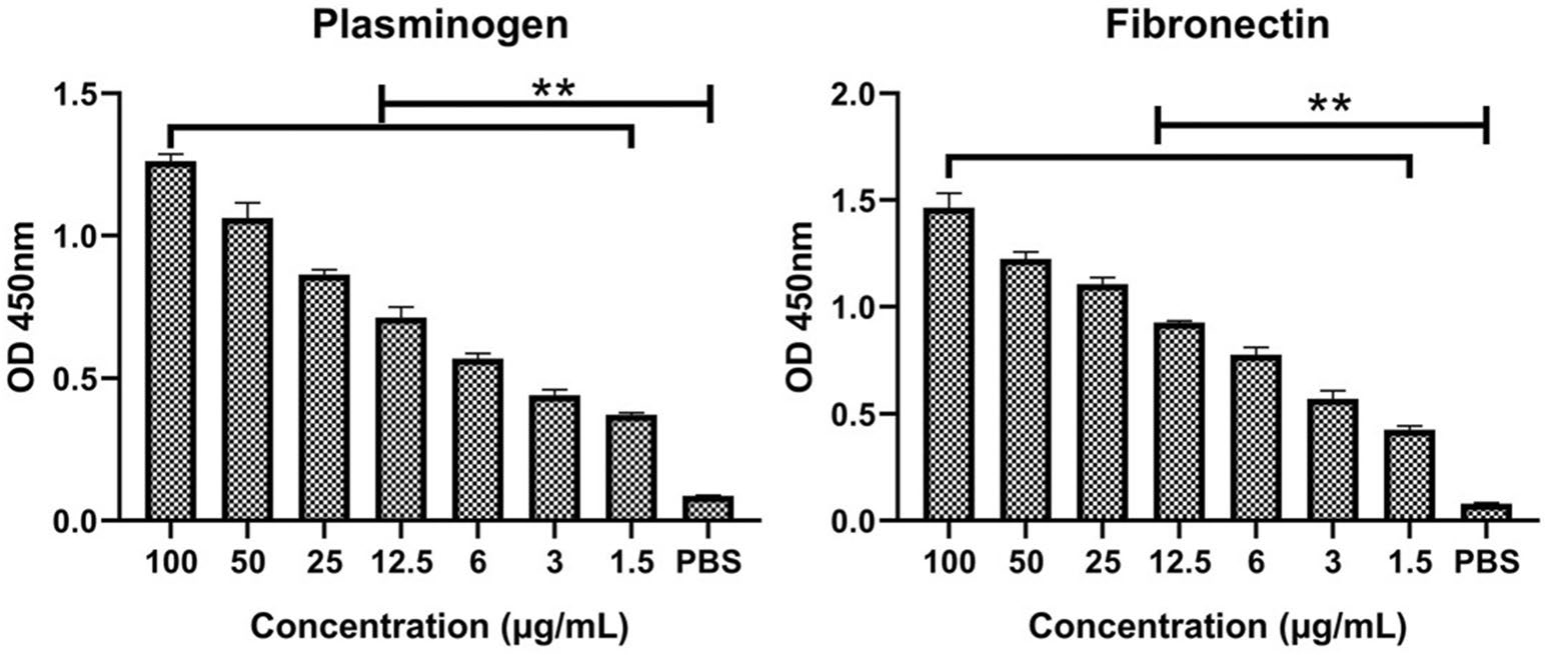
**Ability of *M. hyorhinis*** **enolase to bind plasminogen and fibronectin**. Microtiter plates were coated with plasminogen or fibronectin. Increasing concentrations of rEno protein were added to individual wells. Bound rEno was detected with anti-rEno serum and compared with wells without protein added. The results are expressed as means ± SD of three experiments with triplicate samples (**p < 0.01).

Different concentrations of rEno proteins were placed in 96-well plates coated with either of the two host molecules. After washing, bound rEno was detected by anti-rEno serum. As shown in Figure 3, rEno bound both plasminogen and fibronectin strongly in a dose-dependent manner, while wells not containing rEno show no increase in the OD value at 450 nm.

### Activation of plasminogen bound to enolase

**Figure 4:**
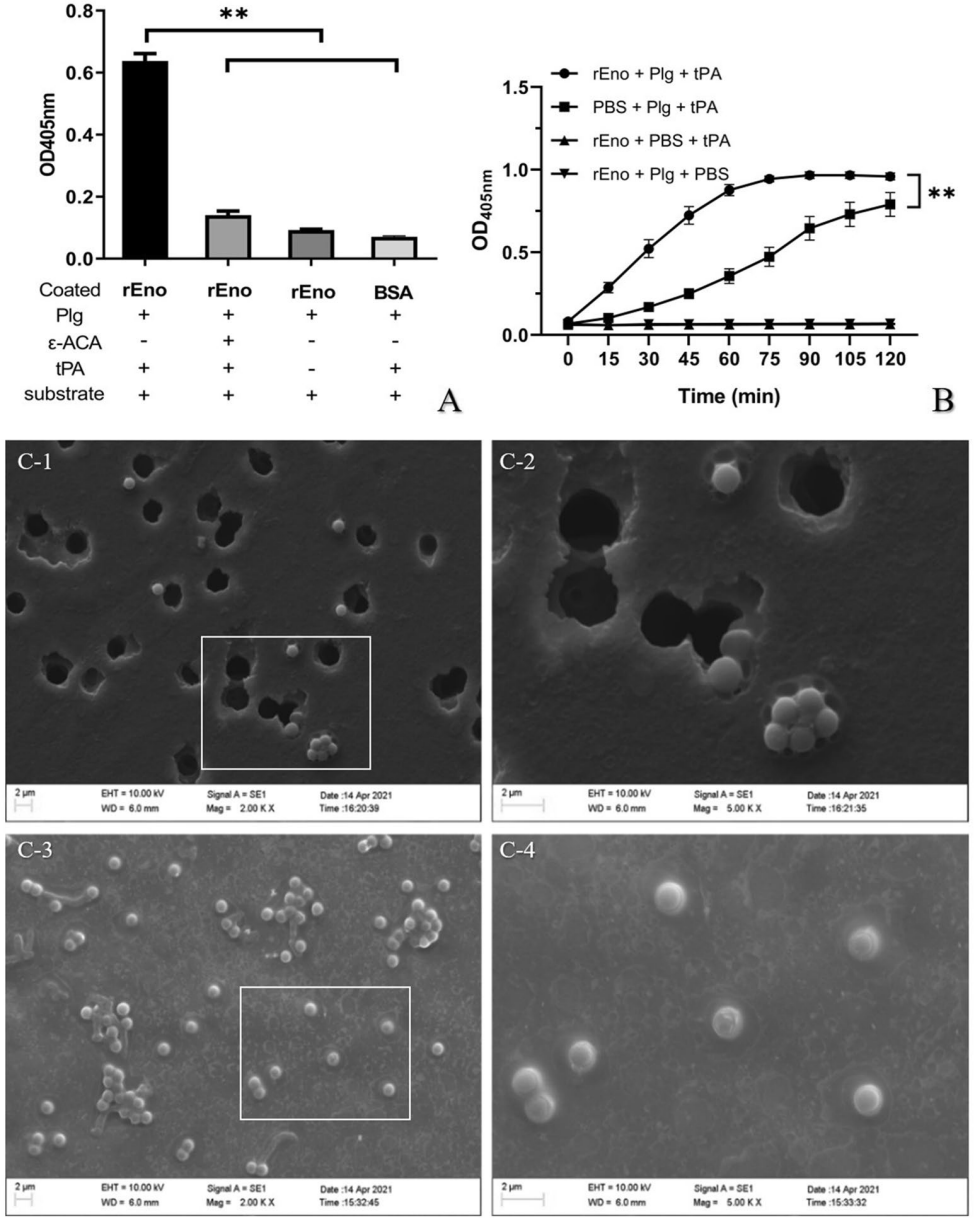
**Activation of plasminogen bound to enolase.** **A** Ability to hydrolyze chemical substrates. Plasminogen was placed in microtiter plates coated with rEno or BSA in the presence or absence of ε-ACA. Bound plasminogen was activated by tPA. A plasmin-specific substrate was added, and the OD value was measured at 405 nm. Wells without tPA served as negative controls. **B** rEno enhances the activation of plasminogen by tPA. Plasminogen was pre-incubated with rEno or PBS prior to the addition of tPA. Activation of plasminogen was measured by adding a plasmin-specific substrate. Wells without Plg or tPA added served as negative controls. **C** Degradation of reconstituted ECM. Matrigel was reconstituted on the surface of the transwell membrane. rEno-harboring or BSA-harboring polystyrene beads, treated with plasminogen and tPA, were added to the upper compartment of the transwell and incubated for 40 h. The surface of transwell membranes was analyzed by SEM. **C-2** and **C-4** are enlarged views of parts of **C-1** and **C-3**, respectively. Results are expressed as means ± SD of three experiments with triplicate samples (**p < 0.01).

Activation of enolase-bound plasminogen by host activator tPA was first detected using a plasmin-specific chromogenic substrate. As shown in Figure 4A, wells coated with rEno proteins and treated with plasminogen and tPA show an increase in the OD value at 405 nm, compared with BSA-coated control wells. Thus, plasminogen bound to coated-rEno was converted to active plasmin. OD values were significantly decreased in wells incubated with ε-ACA in addition, compared with those of wells incubated with only plasminogen and tPA. No increase was observed in the wells incubated with plasminogen but not tPA.

We subsequently explored whether rEno could enhance the activation of plasminogen by tPA. The kinetic curve of plasminogen activation by tPA was measured in the presence and absence of rEno protein (Figure 4B). The rate of activation of plasminogen was enhanced by the presence of rEno, compared to the wells containing only plasminogen and tPA (*p* < 0.01). No increase of OD values was observed in the wells containing only plasminogen with rEno, or rEno with tPA.

In addition to hydrolyzing chemical substrates, we assessed proteolysis of Matrigel, a complex ECM preparation. rEno- or BSA-coated polystyrene beads treated with plasminogen and tPA were placed in the upper compartment of a transwell covered with reconstituted Matrigel. After overnight incubation, changes in Matrigel were assessed by SME. As shown in Figure 4C, incubation of rEno-coated beads treated with plasminogen and tPA resulted in significant damage, characterized by depressions on the ECM network. In some places, the ECM layer was completely degraded, and holes in the transwell membrane could be observed. No damage was observed in control transwells incubated with BSA-coated beads.

### C-terminal lysine residues are critical for enolase to bind plasminogen

**Figure 5:**
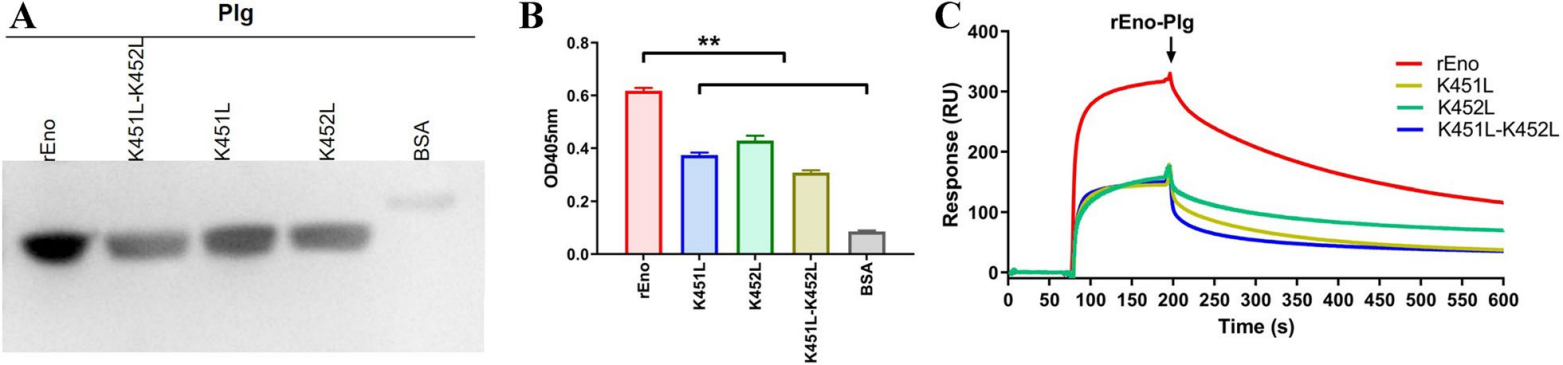
**Interactions of rEno and its mutants with plasminogen.** **A** Far-Western blotting analysis. WT rEno, single mutants K451L and K452L, and double mutant K451L-K452L were transferred to a PVDF membrane and incubated with plasminogen and anti-plasminogen antibody. BSA served as a negative control. Protein bands were visualized using ECL substrate. **B** Activity of bound plasminogen. Plasminogen placed in the microtiter plates coated with WT rEno, each of the three mutants, or BSA. After washing, bound plasminogen was treated with tPA, followed by substrate, and the OD value was measured at 405 nm. Results are expressed as means ± SD of three experiments with triplicate samples (**p < 0.01). **C** SPR analysis. WT rEno and the three mutants were separately injected over immobilized plasminogen. Sensorgrams show the binding of immobilized plasminogen to WT rEno and the three mutants. The arrow indicates the end of the injection period, at which point dissociation of WT rEno and the three mutants from plasminogen can be observed. The different proteins are represented by different colored lines. RU, resonance units.

C-terminal lysine residues in PlgR molecules are usually critical in their interaction with plasminogen. *M. hyorhinis* enolase contains 38 lysines, two of which are located at the C-terminus. These C-terminal lysines were replaced with leucine to generate mutant enolase molecules; two single-site mutants (K451L and K452L) and one double-site mutant (K451L-K452L) were prepared. The interactions of WT and mutant enolases with plasminogen were then compared. The results of far-Western blotting are shown in Figure 5A. rEno specifically bound to plasminogen, but no specific reaction was observed for BSA controls. The gray scales of all three different mutants were lower than for WT enolase, especially in the case of the double-site mutant K451L-K452L. As binding decreased, the ability of plasminogen to hydrolyze substrates decreased markedly when bound to all three mutants (K451L = 39.5%; K452L = 30.5%; K451L-K452L = 50.1%) compared with WT rEno-bound plasminogen (*p* < 0.01; Figure 5B).

Furthermore, SPR analysis was conducted to investigate the kinetics of the interaction and the affinity (Figure 5C). The results indicate that rEno binds to plasminogen with high affinity (dissociation equilibrium constant (*K*_D_) = 1.40 ± 0.07 nM), while all mutants show significantly reduced affinity (*K*_D_ of K451L = 28.01 ± 0.34 nM, *K*_D_ of K452L = 15.81 ± 5.24 nM, *K*_D_ of K451L-K452L = 59.42 ± 7.33 nM, *p* < 0.01). The lowest affinity was measured for the K451L-K452L double mutant.

### Potential role of C-terminal lysine residues in the interaction between enolase and fibronectin

**Figure 6:**
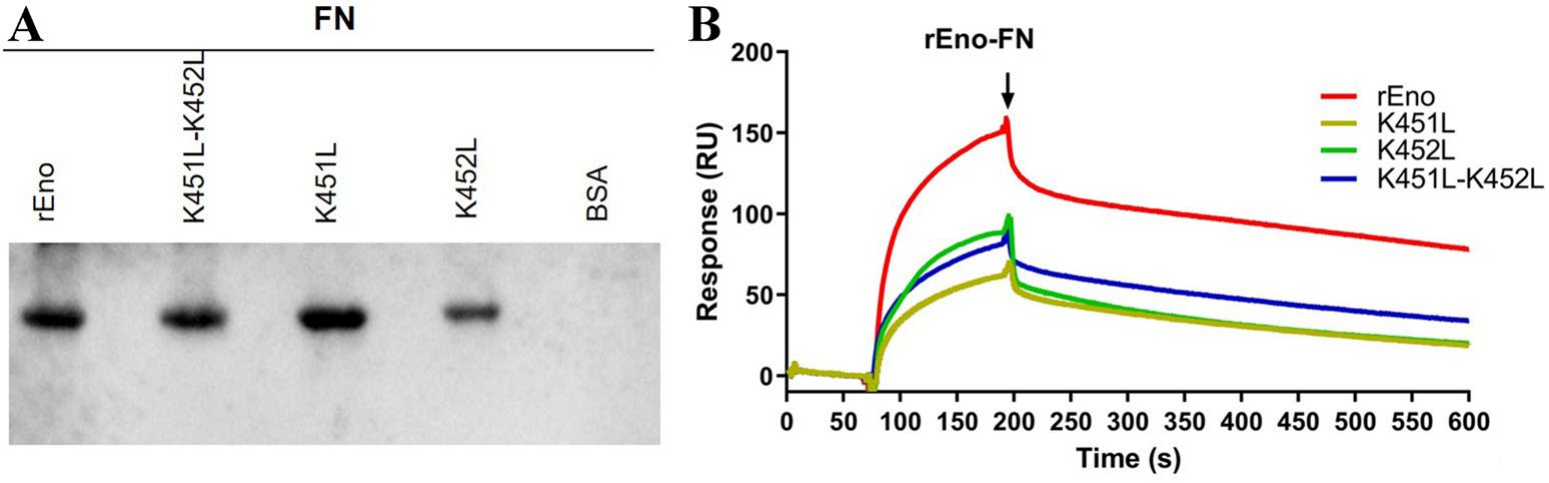
**Interaction of rEno and its mutants with fibronectin.** **A** Far-Western blotting analysis. WT rEno, single mutants K451L and K452L, and double mutant K451L-K452L were transferred to a PVDF membrane and incubated with fibronectin and anti-fibronectin antibody. BSA served as a negative control. Protein bands were visualized using ECL substrate. **B** SPR analysis. rEno and the three different mutants were injected over immobilized fibronectin. Sensorgrams depict the binding of immobilized fibronectin to rEno and the three different mutants. The arrow indicates the end of the injection period, at which point dissociation of rEno and the three different mutants from fibronectin can be observed. Different proteins are represented by different colored lines. RU, resonance units.

Experiments were carried out to investigate the potential role of C-terminal lysine residues in the interaction of enolase with fibronectin. As shown in Figure 6A, the hybridization bands of all mutants showed no significant decrease in gray scales compared with that of WT rEno, except for K452L. However, the SPR results indicate that all three mutants show significantly reduced affinity (*K*_D_ of K451L = 77.9 ± 6.81 nM, *K*_D_ of K452L = 70.49 ± 14.27 nM, *K*_D_ of K451L-K452L = 75.2 ± 1.95 nM, *p* < 0.01), compared with the wild-type enolase for which the *K*_D_ was 14.30 ± 2.26 nM (Figure 6B).

## Discussion

Mycoplasmas are bacteria lacking a cell wall that possess a remarkably compact genome (usually between 700 and 1000 kbp) and engage in long-term interactions with their hosts. Limited genomic resources have favoured the evolution of proteins with multiple functions. Proteins that perform two very different functions, often in two different subcellular locations, are referred to as moonlighting proteins. The first moonlighting protein described was GAPDH on the surface of group A streptococci [17]. Surprisingly, many moonlighting proteins in eukaryotes and prokaryotes are highly conserved metabolic enzymes or molecular chaperones [18]. The compact genome limits the anabolic and catabolic capacity of mycoplasmas compared with other bacteria. They use glycolysis as the major pathway for ATP production via substrate-level phosphorylation. Surface-displayed glycolytic enzymes moonlighting as adhesins or with other functions have been reported for various mycoplasma species, such as *M. pneumoniae* [19], *M. bovis* [20], and *M gallisepticum* [21]. Enolase is assumed to be the core protein in the interactions between all enzymes involved in this pathway [22]. Although lacking canonical extracellular targeting motifs, it can express on the surface of many bacteria including mycoplasmas, and it performs multiple functions [9, 21, 23, 24].

In the present study, flow cytometry and colony immunological hybridization analyses were performed to probe the presence of enolase on the surface of *M. hyorhinis*. Its involvement in cytoadhesin was demonstrated by its adherence to PK-15 cells, as well as inhibition of *M. hyorhinis* adhesion by enolase-specific antibodies. The cytoadhesion function of enolase has been reported in a variety of mycoplasma species, including *M. hyopneumoniae* [25], *M. suis* [26], *M. bovis* [20], and *M. gallisepticum* [21]. The contribution of enolase to the adhesion ability of *M. hyorhinis* is considered indispensable because adhesion to PK-15 cells was decreased by ~ 60% when enolase on the bacterial surface was blocked by antibodies. However, there must be other adhesion molecules functioning together, since inhibition by anti-enolase antibodies was only partial.

In addition to binding cell membrane proteins, interactions of enolase with other host molecules were also investigated. A fascinating feature of moonlighting proteins is that their moonlighting functions are somewhat similar. The most common interaction partner proteins of hosts are plasminogen and ECM proteins [27]. Plasminogen is the central component of the fibrinolytic system, a tightly-controlled broad-spectrum proteolytic system. The fibrinolytic system plays an important role in several physiological processes including degradation of fibrin clots, and various ECM and connective tissue components [28]. Many invasive bacteria can utilize the fibrinolytic system to disperse from their initial site of colonization and gain entry to distal tissue sites by secreting plasminogen activators or expressing plasminogen receptors on their surface. The latter method is more universal and bacterial surface-bound plasminogen is transformed into plasmin by host plasminogen activators, hence bacteria can turn themselves into proteolytic organisms by hijacking the host-derived fibrinolytic system [29]. Plasmin can also activate latent matrix metalloproteinases to further enhance the hydrolysis of ECM. *M. hyorhinis* is a frequent inhabitant of the porcine respiratory tract, where it can cause serofibrinous inflammation of serous body cavities and joints in piglets, including polyserositis, arthritis, eustachitis, otitis, conjunctivitis, meningoencephalitis, and pneumonia [1]. This indicates that *M. hyorhinis* may breach tissue barriers in vivo, which may be critical for disease establishment and progression. However, the exact details remain poorly understood. In previous work, we demonstrated GAPDH expression on the surface of *M. hyorhinis*, and showed that it acts as a PlgR to bind plasminogen, and thereby help bacteria to degrade ECM [8]. Indeed, glycolytic enzymes are important bacterial PlgR members, among which enolase and GAPDH are the most well-known. The role of *M. hyorhinis* enolase in plasminogen hijacking was investigated in the current study, and a strong interaction between plasminogen and rEno was observed, with a *K*_D_ value of 1.40 nM. The rEno-bound plasminogen could be activated by tPA to form plasmin, an active serine protease, that degraded a specific substrate and a reconstituted ECM gel. These results suggest that enolase is another important PlgR in *M. hyorhinis*.

Plasminogen consists of five different homologous kringle domains (K1 − K5) with lysine-binding sites that facilitate interaction with target molecules and PlgR. It has been reported that plasminogen bound to PlgR may undergo conformational changes that enhance its susceptibility to activation by plasminogen activators [30–32]. An enhancement in plasminogen activation by tPA in the presence of rEno was observed, indicating the occurrence of a conformational change following binding.

Lysine residues of PlgR, especially the C-terminal lysines, typically play core roles in the interaction with plasminogen [33, 34]. Activation of rEno-bound plasminogen was significantly inhibited by the lysine analogue ε-ACA (Figure 4), which verified the importance of the lysine residues in the interaction of *M. hyorhinis* enolase with plasminogen. As shown in Figure 5, mutation of both Lys451 and Lys452 decreased the binding of *M. hyorhinis* enolase for plasminogen, suggesting an important role for these C-terminal lysines. The K451L-K452L double-site mutant exhibited the lowest activity and affinity. This indicates that both lys451 and lys452 are important for *M. hyorhinis* enolase to bind plasminogen. However, replacement of both C-terminal lysines did not completely abolish binding; the K451L-K452L double-site mutant retained considerable ability to bind plasminogen, with a *K*_D_ of 59.42 ± 7.33 nM. This suggests the existence of other internal binding sites. In streptococcal surface enolase, an internal plasminogen-binding site comprising nine residues mediates binding to plasminogen, in concert with the terminal lysine residues [35]. Similarly, another study showed that binding of Group A Streptococcus surface enolase to human plasminogen is mediated by two internal lysines (252 and 255) in addition to the C-terminal lysines [36]. Knaust et al. reported that internal lysine residues (rather than a terminal lysine) are important for plasminogen binding by *Neisseria meningitides* enolase, DnaK, and peroxiredoxin [37]. The plasminogen-binding abilities of different regions of *M. hyorhinis* enolase and the key amino acids of it will be further studied in future work.

In addition to regulating coagulation, bacterial hijacking of plasminogen/plasmin affects innate immunity in multiple ways. Plasminogen can bind C3, C3b, C3d, and C5, and cleave C3b and C5 when active as plasmin. Cleavage of C3b and C5 inhibits the activity of the complement system [38]. Plasmin can also cleave the hinge region of IgG bound to bacteria, and removal of the Fc fragment leads to decreased phagocytosis by macrophages [39, 40]. Therefore, PlgR likely plays an important role in the persistence of *M. hyorhinis* infection.

Like plasminogen, fibronectin is another host molecule that often interacts with mycoplasma moonlighting surface proteins [41–43]. A strong interaction between *M. hyorhinis* enolase and fibronectin was also observed in the present study. Fibronectin is a high-molecular-weight glycoprotein component of the ECM that mediates a wide variety of cellular interactions, and plays important roles in cell adhesion, migration, growth, and differentiation [44]. Many bacteria utilize fibronectin and other ECM molecules to strengthen cytoadhesion [45–47]. In previous work, fibronectin was found to be abundant in the ciliary borders of the airway epithelium [43], a place where *M. hyorhinis* is most commonly detected alongside tonsils [48]. Binding to fibronectin likely facilitates adherence to and colonization in the ciliary epithelium of the respiratory tract. Invasion of infected cells by *M. hyorhinis* has been reported in various studies [49, 50]. It is an important mechanism of evasion of the host immune system. Fibronectin contains an Arg-Gly-Asp (RGD) sequence that binds to integrins on the surface of host cells, through which fibronectin serves as a molecular bridge between bacterial adhesion and integrins on the host cell surface, and promotes the cytoskeletal rearrangements required for internalization via an integrin-dependent signaling pathway [51]. Therefore, interaction of enolase with fibronectin is also likely to participate in multiple processes of *M. hyorhinis* infection. In the present study, we were surprised to discover that binding affinities of the C-terminal lysine enolase mutants to fibronectin was also reduced compared with WT enolase, although the difference in far-Western blotting was not significant (Figure 6). It indicates a potential role of C-terminal lysine in the interaction between *M. hyorhinis* enolase and fibronectin. The function of lysines in the interaction with fibronectin has not been confirmed yet to our knowledge. It has been reported that the enolase of *Streptococcus suis* binds to fibronectin in a lysine-dependent manner, indicated by a competitive inhibition by ε-ACA [52].

A growing number of bacterial moonlighting proteins engage in more than one moonlighting activity. Enolase of *Streptococcus pneumoniae* reportedly binds human complement inhibitor C4b-binding protein and contributes to complement evasion [53]. Enolase from *Aspergillus fumigatus* can bind four complement regulators including factor H, factor-H-like protein 1, C4b-binding protein, and plasminogen [23]. Interactions between *Candida* enolase and another complement inhibitor (vitronectin) have also been reported [54]. The potential role of *M. hyorhinis* enolase in interactions with complement regulators and escaping host immunity are intriguing topics to be investigated in the future.

In conclusion, we revealed that *M. hyorhinis* enolase is a multifunctional protein on the bacterial surface. It participates in cytoadhesion, and also functions as a receptor for two important host molecules (plasminogen and fibronectin). C-terminal lysines of enolase play important roles in these interactions. The findings suggest that enolase plays an indispensable role in infection and systemic invasion in *M. hyorhinis*.

## Supplementary Information

**Additional file 1**. **Assessment of the specificity of the prepared polyclonal antibody against** ***M. hyorhinis*** **enolase.** (**A**) The whole cell lysate of *M. hyorhinis* and purified WT rEno protein were subjected to 12% SDS-PAGE and transferred to a polyvinylidene fluoride (PVDF) membrane. After blocking with 5% skim milk in TBST buffer, the membrane was incubated with the anti-rEno serum (1:5000 dilution), followed by horseradish peroxidase (HRP)-conjugated goat anti-rabbit IgG (1:10 000 dilution). Finally, filters were developed with Electro-Chemi-Luminescence (ECL) substrate using a ChemiDoc XRS+ system (Bio-Rad, USA). (**B**) The serum obtained before immunization was used as the negative control. M, protein molecular weight marker; lane 1, whole cell lysate of *M. hyorhinis*; lane 2, purified WT rEno.

## Data Availability

The datasets supporting the conclusions of this article are included within the article and its additional files.

## Abbreviations

BM: basement membrane
BSA: bovine serum albumin
CCU: color change unit
ECM: extracellular matrix
ELISA: enzyme-linked immunosorbent assay
Eno: enolase
GAPDH: glyceraldehyde-3-phosphate dehydrogenase
MFI: mean fluorescence intensity
MPAA: micro titer plate adhesion assay
PBS: phosphate buffered saline
PCR: polymerase chain reaction
Plg: plasminogen
PlgR: plasminogen receptor
rEno: recombinant enolase
SPR: surface plasmon resonance
tPA: tissue plasminogen activator
Vlp: variable lipoprotein
WT: wild type
ε-ACA: ε-aminocaproic acid

## References

[1] Pieters M, Maes D, Zimmermann JJ, Karriker LA, Ramirez A, Schwartz KJ, Stevenson GW, Zhang J (2019). Mycoplasmosis. Diseases of swine.

[2] Morita T, Ohiwa S, Shimada A, Kazama S, Yagihashi T, Umemura T (1999). Intranasally inoculated *Mycoplasma hyorhinis* causes eustachitis in pigs. Vet Pathol.

[3] Martinson B, Minion FC, Jordan D (2018). Development and optimization of a cell-associated challenge model for *Mycoplasma hyorhinis* in 7-week-old cesarean-derived, colostrum-deprived pigs. Can J Vet Res.

[4] Huang S, Li JY, Wu J, Meng L, Shou CC (2001). Mycoplasma infections and different human carcinomas. World J Gastroenterol.

[5] Vande Voorde J, Balzarini J, Liekens S (2014). Mycoplasmas and cancer: focus on nucleoside metabolism. EXCLI J.

[6] Duan H, Chen L, Qu L, Yang H, Song SW, Han Y, Ye M, Chen W, He X, Shou C (2014). *Mycoplasma hyorhinis* infection promotes NF-kappaB-dependent migration of gastric cancer cells. Cancer Res.

[7] Xiong Q, Wang J, Ji Y, Ni B, Zhang B, Ma Q, Wei Y, Xiao S, Feng Z, Liu M, Shao G (2016). The functions of the variable lipoprotein family of *Mycoplasma hyorhinis* in adherence to host cells. Vet Microbiol.

[8] Wang J, Li Y, Pan L, Li J, Yu Y, Liu B, Zubair M, Wei Y, Pillay B, Olaniran AO, Chiliza TE, Shao G, Feng Z, Xiong Q (2021). Glyceraldehyde-3-phosphate dehydrogenase (GAPDH) moonlights as an adhesin in *Mycoplasma hyorhinis* adhesion to epithelial cells as well as a plasminogen receptor mediating extracellular matrix degradation. Vet Res.

[9] Bergmann S, Rohde M, Chhatwal GS, Hammerschmidt S (2001). alpha-Enolase of *Streptococcus pneumoniae* is a plasmin(ogen)-binding protein displayed on the bacterial cell surface. Mol Microbiol.

[10] Serek P, Bednarz-Misa I, Pietkiewicz J, Dudek B, Mierzchala-Pasierb M, Jermakow K, Drab M, Gamian A (2020). *Salmonella* Typhimurium enolase-like membrane protein is recognized by antibodies against human enolase and interacts with plasminogen. Adv Clin Exp Med.

[11] Rahi A, Matta SK, Dhiman A, Garhyan J, Gopalani M, Chandra S, Bhatnagar R (2017). Enolase of *Mycobacterium tuberculosis* is a surface exposed plasminogen binding protein. Biochim Biophys Acta Gen Subj.

[12] Serek P, Lewandowski L, Dudek B, Pietkiewicz J, Jermakow K, Kapczynska K, Krzyzewska E, Bednarz-Misa I (2021). *Klebsiella pneumoniae* enolase-like membrane protein interacts with human plasminogen. Int J Med Microbiol.

[13] Ayon-Nunez DA, Fragoso G, Espitia C, Garcia-Varela M, Soberon X, Rosas G, Laclette JP, Bobes RJ (2018). Identification and characterization of *Taenia solium* enolase as a plasminogen-binding protein. Acta Trop.

[14] Vanegas G, Quinones W, Carrasco-Lopez C, Concepcion JL, Albericio F, Avilan L (2007). Enolase as a plasminogen binding protein in *Leishmania mexicana*. Parasitol Res.

[15] Guo Y, Zhu H, Wang J, Huang J, Khan FA, Zhang J, Guo A, Chen X (2017). TrmFO, a fibronectin-binding adhesin of *Mycoplasma bovis*. Int J Mol Sci.

[16] Fourour S, Fablet C, Tocqueville V, Dorenlor V, Eono F, Eveno E, Kempf I, Marois-Crehan C (2018). A new multiplex real-time TaqMan((R)) PCR for quantification of *Mycoplasma hyopneumoniae*, *M. hyorhinis* and *M. flocculare*: exploratory epidemiological investigations to research mycoplasmal association in enzootic pneumonia-like lesions in slaughtered pigs. J Appl Microbiol.

[17] Pancholi V, Fischetti VA (1992). A major surface protein on group A streptococci is a glyceraldehyde-3-phosphate-dehydrogenase with multiple binding activity. J Exp Med.

[18] Henderson B (2014). An overview of protein moonlighting in bacterial infection. Biochem Soc Trans.

[19] Grundel A, Pfeiffer M, Jacobs E, Dumke R (2015). Network of surface-displayed glycolytic enzymes in *Mycoplasma pneumoniae* and their interactions with human plasminogen. Infect Immun.

[20] Song Z, Li Y, Liu Y, Xin J, Zou X, Sun W (2012). Alpha-Enolase, an adhesion-related factor of *Mycoplasma bovis*. PLoS ONE.

[21] Chen H, Yu S, Shen X, Chen D, Qiu X, Song C, Ding C (2011). The *Mycoplasma gallisepticum* alpha-enolase is cell surface-exposed and mediates adherence by binding to chicken plasminogen. Microb Pathog.

[22] Dutow P, Schmidl SR, Ridderbusch M, Stulke J (2010). Interactions between glycolytic enzymes of *Mycoplasma pneumoniae*. J Mol Microbiol Biotechnol.

[23] Dasari P, Koleci N, Shopova IA, Wartenberg D, Beyersdorf N, Dietrich S, Sahagun-Ruiz A, Figge MT, Skerka C, Brakhage AA, Zipfel PF (2019). Enolase from aspergillus fumigatus is a moonlighting protein that binds the human plasma complement proteins factor H, FHL-1, C4BP, and plasminogen. Front Immunol.

[24] Berry IJ, Jarocki VM, Tacchi JL, Raymond BBA, Widjaja M, Padula MP, Djordjevic SP (2017). N-terminomics identifies widespread endoproteolysis and novel methionine excision in a genome-reduced bacterial pathogen. Sci Rep.

[25] Chen R, Yu Y, Feng Z, Gan R, Xie X, Zhang Z, Xie Q, Wang W, Ran T, Zhang W, Xiong Q, Shao G (2019). Featured species-specific loops are found in the crystal structure of mhp eno, a cell surface adhesin from *Mycoplasma hyopneumoniae*. Front Cell Infect Microbiol.

[26] Schreiner SA, Sokoli A, Felder KM, Wittenbrink MM, Schwarzenbach S, Guhl B, Hoelzle K, Hoelzle LE (2012). The surface-localised alpha-enolase of *Mycoplasma suis* is an adhesion protein. Vet Microbiol.

[27] Franco-Serrano L, Sanchez-Redondo D, Najar-Garcia A, Hernandez S, Amela I, Perez-Pons JA, Pinol J, Mozo-Villarias A, Cedano J, Querol E (2021). Pathogen moonlighting proteins: from ancestral key metabolic enzymes to virulence factors. Microorganisms.

[28] Law RH, Abu-Ssaydeh D, Whisstock JC (2013). New insights into the structure and function of the plasminogen/plasmin system. Curr Opin Struct Biol.

[29] Raymond BB, Djordjevic S (2015). Exploitation of plasmin(ogen) by bacterial pathogens of veterinary significance. Vet Microbiol.

[30] Lahteenmaki K, Edelman S, Korhonen TK (2005). Bacterial metastasis: the host plasminogen system in bacterial invasion. Trends Microbiol.

[31] Seymour LM, Jenkins C, Deutscher AT, Raymond BB, Padula MP, Tacchi JL, Bogema DR, Eamens GJ, Woolley LK, Dixon NE, Walker MJ, Djordjevic SP (2012). Mhp182 (P102) binds fibronectin and contributes to the recruitment of plasmin(ogen) to the *Mycoplasma hyopneumoniae* cell surface. Cell Microbiol.

[32] Peetermans M, Vanassche T, Liesenborghs L, Lijnen RH, Verhamme P (2016). Bacterial pathogens activate plasminogen to breach tissue barriers and escape from innate immunity. Crit Rev Microbiol.

[33] Aljannat MAK, Oldfield NJ, Albasri HM, Dorrington LKG, Ohri RL, Wooldridge KG, Turner DPJ (2020). The moonlighting peroxiredoxin-glutaredoxin in *Neisseria meningitidis* binds plasminogen via a C-terminal lysine residue and contributes to survival in a whole blood model. Microb Pathog.

[34] Derbise A, Song YP, Parikh S, Fischetti VA, Pancholi V (2004). Role of the C-terminal lysine residues of streptococcal surface enolase in Glu- and Lys-plasminogen-binding activities of group A streptococci. Infect Immun.

[35] Ehinger S, Schubert WD, Bergmann S, Hammerschmidt S, Heinz DW (2004). Plasmin(ogen)-binding alpha-enolase from *Streptococcus pneumoniae*: crystal structure and evaluation of plasmin(ogen)-binding sites. J Mol Biol.

[36] Cork AJ, Jergic S, Hammerschmidt S, Kobe B, Pancholi V, Benesch JLP, Robinson CV, Dixon NE, Aquilina JA, Walker MJ (2009). Defining the structural basis of human plasminogen binding by streptococcal surface enolase. J Biol Chem.

[37] Knaust A, Weber MV, Hammerschmidt S, Bergmann S, Frosch M, Kurzai O (2007). Cytosolic proteins contribute to surface plasminogen recruitment of *Neisseria meningitidis*. J Bacteriol.

[38] Barthel D, Schindler S, Zipfel PF (2012). Plasminogen is a complement inhibitor. J Biol Chem.

[39] Rooijakkers SH, van Wamel WJ, Ruyken M, van Kessel KP, van Strijp JA (2005). Anti-opsonic properties of staphylokinase. Microbes Infect.

[40] Vieira ML, de Morais ZM, Vasconcellos SA, Romero EC, Nascimento AL (2011). In vitro evidence for immune evasion activity by human plasmin associated to pathogenic *Leptospira interrogans*. Microb Pathog.

[41] Bao S, Guo X, Yu S, Ding J, Tan L, Zhang F, Sun Y, Qiu X, Chen G, Ding C (2014). *Mycoplasma synoviae* enolase is a plasminogen/fibronectin binding protein. BMC Vet Res.

[42] Huang J, Zhu H, Wang J, Guo Y, Zhi Y, Wei H, Li H, Guo A, Liu D, Chen X (2019). Fructose-1,6-bisphosphate aldolase is involved in *Mycoplasma bovis* colonization as a fibronectin-binding adhesin. Res Vet Sci.

[43] Yu Y, Wang H, Wang J, Feng Z, Wu M, Liu B, Xin J, Xiong Q, Liu M, Shao G (2018). Elongation factor thermo unstable (ef-tu) moonlights as an adhesin on the surface of *Mycoplasma hyopneumoniae* by binding to fibronectin. Front Microbiol.

[44] Pankov R, Yamada KM (2002). Fibronectin at a glance. J Cell Sci.

[45] Yavlovich A, Rottem S (2007). Binding of host extracellular matrix proteins to *Mycoplasma fermentans* and its effect on adherence to, and invasion of HeLa cells. FEMS Microbiol Lett.

[46] Grundel A, Jacobs E, Dumke R (2016). Interactions of surface-displayed glycolytic enzymes of *Mycoplasma pneumoniae* with components of the human extracellular matrix. Int J Med Microbiol.

[47] Casillas-Ituarte NN, Staats AM, Lower BH, Stoodley P, Lower SK (2021). Host blood proteins as bridging ligand in bacterial aggregation as well as anchor point for adhesion in the molecular pathogenesis of *Staphylococcus aureus* infections. Micron.

[48] Gois M, Pospisil Z, Cerny M, Mrva V (1971). Production of pneumonia after intransal inoculation of gnotobiotic piglets with three strains of *Mycoplasma hyorhinis*. J Comp Pathol.

[49] Kornspan JD, Tarshis M, Rottem S (2010). Invasion of melanoma cells by *Mycoplasma hyorhinis*: enhancement by protease treatment. Infect Immun.

[50] Kornspan JD, Tsur M, Tarshis M, Rottem S, Brenner T (2015). *Mycoplasma hyorhinis* induces proinflammatory responses in mice lymphocytes. J Basic Microbiol.

[51] Scibelli A, Roperto S, Manna L, Pavone LM, Tafuri S, Della Morte R, Staiano N (2007). Engagement of integrins as a cellular route of invasion by bacterial pathogens. Vet J.

[52] Esgleas M, Li Y, Hancock MA, Harel J, Dubreuil JD, Gottschalk M (2008). Isolation and characterization of alpha-enolase, a novel fibronectin-binding protein from *Streptococcus suis*. Microbiology.

[53] Agarwal V, Hammerschmidt S, Malm S, Bergmann S, Riesbeck K, Blom AM (2012). Enolase of *Streptococcus pneumoniae* binds human complement inhibitor C4b-binding protein and contributes to complement evasion. J Immunol.

[54] Satala D, Satala G, Karkowska-Kuleta J, Bukowski M, Kluza A, Rapala-Kozik M, Kozik A (2020). Structural insights into the interactions of candidal enolase with human vitronectin, Fibronectin and Plasminogen. Int J Mol Sci.

