# Supplementary figures and images for: A multifunctional enolase mediates cytoadhesion and interaction with host plasminogen and fibronectin in Mycoplasma hyorhinis
Source: Vet Res. 2022 Mar 25;53:26. doi: 10.1186/s13567-022-01041-0 (PMC8951703; doi:10.1186/s13567-022-01041-0)

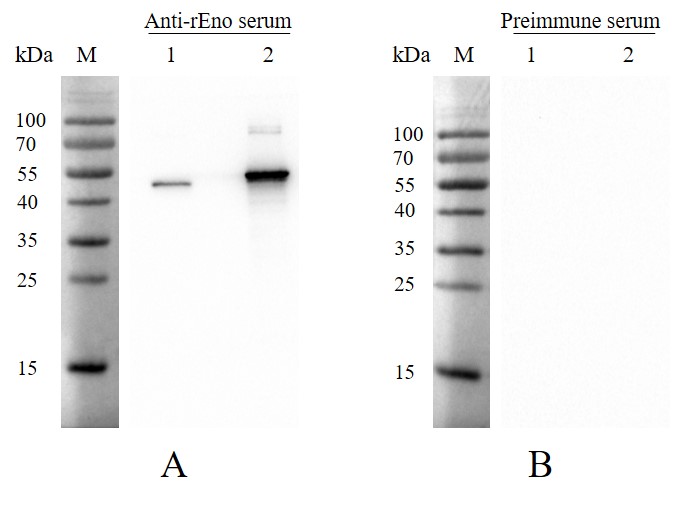

Supplement: Supplementary file 1 — Additional file 1. Assessment of the specificity of the prepared polyclonal antibody against M. hyorhinis enolase. (A) The whole cell lysate of M. hyorhinis and purified WT rEno protein were subjected to 12% SDS-PAGE and transferred to a polyvinylidene fluoride (PVDF) membrane. After blocking with 5% skim milk in TBST buffer, the membrane was incubated with the anti-rEno serum (1:5000 dilution), followed by horseradish peroxidase (HRP)-conjugated goat anti-rabbit IgG (1:10 000 dilution). Finally, filters were developed with Electro-Chemi-Luminescence (ECL) substrate using a ChemiDoc XRS+ system (Bio-Rad, USA). (B) The serum obtained before immunization was used as the negative control. M, protein molecular weight marker; lane 1, whole cell lysate of M. hyorhinis; lane 2, purified WT rEno. [file 13567_2022_1041_MOESM1_ESM.docx]
